# Implications of cognitive and daily living capabilities on early type 2 diabetes management: a preliminary case–control study

**DOI:** 10.1186/s40001-024-01925-1

**Published:** 2024-06-18

**Authors:** Romina Mahmoudi, Farzin Kamari, Reza Naghdi Sadeh, Amirreza Naseri, Vahideh Sadra

**Affiliations:** 1 Endocrine Research Center, Tabriz University of Medical Sciences, Tabriz, Iran; 2 Department of Neurophysiology, Institute of Physiology, Eberhard Karls University of Tübingen, Tübingen, Germany; 3 Research Center of Psychiatry and Behavioral Sciences, Tabriz University of Medical Sciences, Tabriz, Iran; 4 Student Research Committee, Tabriz University of Medical Sciences, Tabriz, Iran; 5 Research Center for Evidence-Based Medicine, Iranian EBM Center: A Joanna Briggs Institute Center of Excellence, Tabriz University of Medical Sciences, Tabriz, Iran

**Keywords:** Diabetes mellitus, Treatment adherence, Dementia, Montreal cognitive assessment, Instrumental activities of daily living

## Abstract

**Background:**

Adherence to the transition from oral agents to insulin injections in Type 2 Diabetes Mellitus therapy varies among patients and is not uniformly successful, leading to suboptimal glycemic control in certain cases. This study aims to investigate the potential correlation between cognitive and daily functional capabilities and glycemic control in middle-aged to older adults (40–74 years old) diagnosed with Type 2 Diabetes Mellitus for less than 10 years, specifically those who have recently transitioned to insulin injections and have lower education levels within the context of a developing country.

**Methods:**

A case–control study was conducted with 30 poorly controlled diabetes mellitus (PCDM) patients recognized by HbA1c levels > 8% compared to 30 fairly controlled diabetes mellitus (FCDM) patients with HbA1c levels ≤ 8%. Basic Montreal Cognitive Assessment (MoCA-B) score of less than 27 was investigated as the exposure among two groups. Additionally, intra- and inter-battery correlations were assessed among MoCA-B and Instrumental Activities of Daily Living (IADL) domains using Pearson’s *r*.

**Results:**

The primary outcomes showed no crude difference between MoCA-B scores in the two diabetic groups (*p*-value = 0.82). However, after adjusting for age, education, and IADL scores, cognitive decline in the less-educated younger elderly with high IADL scores demonstrated an unexpected protective effect against PCDM (*p*-value < 0.0001, OR 95% CI = 0–0.26). In linear regression analysis among MoCA-B and IADL scores, “delayed recall” and “orientation” domains from MoCA-B, and “managing medications” and “using the phone” from IADL were negatively associated with HbA1c levels (*p*-values of < 0.01, 0.043, 0.015, and 0.023, respectively). Intra- and inter-battery correlations further illustrated a strong association between MoCA-B’s “orientation” with IADL’s “using the phone” and “managing medications” (*p*-values < 0.0001).

**Conclusion:**

Superior performance in certain cognitive domains is linked to better glycemic control. Still, since assessing cognitive domains may be timely in clinical routine, a potential rapid approach might be taken by assessing patients’ instrumental abilities to use cell phone or manage medications. Future studies including a larger sample size and a broader spectrum of psychosocial factors are needed to elaborate on our findings.

## Background

Diabetes mellitus (DM) is a group of metabolic syndromes that are characterized by high blood glucose levels resulting from deficits in insulin-related pathways [1]. In Type 2 DM (T2DM), which accounts for more than 90% of persons with diabetes, the resistance of the insulin receptors on hepatic and muscular cells is the main reason for increased blood levels of glucose [2]. In 2021, approximately 537 million adults had DM worldwide, and by projecting trends for overweight and obesity, it has been estimated that the global DM prevalence would upsurge by 783.2 million in 2045 [3]. Globally, DM is the ninth leading cause of death [4] and the first cause of new cases of vision loss among the 20–74 age interval [5]. DM is traditionally associated with a wide range of macro and microvascular complications, while a set of emerging complications, like DM-associated cognitive decline and functional disability, are drawing more attention in the recent literature [6]. The effects of DM on the cognitive decline are also well studied in the literature [7–9]; see a review in [6]. A gradual and chronic decline in cognitive function is commonly believed to occur which particularly accelerates with the aging process. In the large-scale cohort study of Whitehall II, it has been demonstrated that cognitive decline in the middle-aged to older adults with a recent diagnosis of T2DM (5 to 10 years) is negligible [10]. This highlights the importance of age in determining the extent of cognitive decline in individuals with T2DM. Besides age, maintaining tight control of glycemic levels is also crucial in preventing cognitive decline. In general, comprehensive treatment strategies are believed to be crucial in controlling the early- and late-onset complications, and the cognitive decline is of no exception.

Success in comprehensive management of DM is heavily dependent on the medication compliance and adherence of patients [11]. Patients demonstrate different levels of compliance with their anti-diabetes medications, and this might at some degree be related to their level of knowledge, education, cognition and alertness [12]. The core objective of our study arises from a recurring challenge observed in the endocrinological wards, where transitioning middle-aged to older patients from oral agents to insulin injections [13] results in deteriorating glycemic control due to a lesser degree of adherence. This necessitated our focus on middle-aged to older individuals with less than 10 years since the onset of T2DM, so-called recently diagnosed T2DM, a population wherein diabetes-related cognitive decline is improbable within the clinical and pathological contexts, as indicated by existing literature [10]. Although the level of education is plausibly a crucial factor in therapy compliance, studies which are examining the correlation between cognitive function, insulin adherence, and glycemic control, particularly in low education populations, are notably lacking in the literature. In this study, we aimed to investigate the potential correlation between cognitive and daily functional capabilities and glycemic control in middle-aged to older adults diagnosed with Type 2 Diabetes Mellitus for less than 10 years, specifically those who have recently transitioned to insulin injections and have lower education levels within the context of a developing country.

## Methods

### Study design

The current study was designed as a case–control investigation to examine the association between cognitive and instrumental abilities, and T2DM management in a minimally literate population. The cases were distinguished from the controls with an HbA1c of more than 8%. The variables under investigation among cases and controls were the Basic Montreal Cognitive Assessment (MoCA-B), Activities of Daily Living (ADL), and Instrumental Activities of Daily Living (IADL) scores. Age, gender, literacy, and other demographic factors were controlled as covariates. The data collection spanned 15 months starting from November 2019, with an 8-month pause due to the COVID-19 emergency, resulting in an effective recruitment period of 7 months. Sample size was derived from the variables of a similar study on Iranian population [14].

### Study population

60 T2DM patients were recruited into the study, separated into two groups of 30 cases and 30 controls. The inclusion criteria were (1) the diagnosis criteria of T2DM according to the criteria established by the American Diabetes Association (ADA) [1]; (2) the age of patients to be ≥ 40 and < 75 years; (3) the duration of diagnosed T2DM less than 10 years; (4) a transition from oral agents to a regular insulin injection therapy in the past 3 to 6 months, i.e., *recent* transition. In other words, patients transitioned from oral agents to insulin injections with an indication due to antidiabetic agent failure, three to six months before recruitment in the study. Recruitment took place during a follow-up visit following the transition, at which point patients were categorized into cases and controls depending on their HbA1c levels at recruitment. The exclusion criteria were (1) drug or alcohol abuse or dependence; (2) cerebral stroke or other neurological conditions; (3) depression; (4) use of possible or known cognitive-impairing drugs in the previous month; (5) acute coronary syndrome; (6) decompensated heart failure; (7) severe renal dysfunction (serum creatinine > 2.5 mg/dL); (8) active malignancy; (9) active infection; (10) chronic liver failure; (11) body mass index (BMI) ≥ 35; (12) autoimmune diseases, and (13) any surgical procedure related to DM. It is to be noted that all included patients were middle-aged or younger at the time of their diagnosis. The cases entailed patients with an HbA1c measurement equal to or more than 8%, (183 mg/dL or 10.2 mmol/L). Controls were T2DM patients with the same criteria as above who had an HbA1c measurement equal to or less than 8%, i.e. fairly controlled T2DM [15, 16]. Patients were recruited from the Imam-Reza University Hospital, Tabriz University of Medical Sciences, Tabriz, Iran.

### Cognitive and daily activity assessment

For the cognitive assessment, the standardized translated version of the MoCA-B battery [17] was performed on all patients via a specifically-trained and dedicated medical practitioner. The selection of MoCA-B among other cognitive screening tests [18] was due to its excellent sensitivity [19], availability of a validated translated version in Farsi [20], and our existing expertise in performing it during the clinical visits. Furthermore, to evaluate the extent of daily life activity and physical disabilities, ADL and IADL [21] batteries were performed by the same evaluator. Both ADL and IADL have been previously described as valuable assessment tools in the context of DM [22, 23]. Patients with low scores of ADL or IADL due to non-cognitive etiologies, e.g., musculoskeletal restrictions, were subsequently excluded from the study. All tests were performed at once in a calm environment and the test results were concurrently documented in an Excel sheet.

### Blood sampling and HbA1c measurement

Blood samples were collected on the next day of each interview. Serum separator tubes were used for creatinine level assessments, and Ethylenediaminetetraacetic acid (EDTA) tubes were used for complete blood count (CBC) and HbA1c measurements. The blood samples were obtained in the morning after an 8-h of continuous fasting. HbA1c was measured using immuno-turbidimetry methodology enhanced by latex particles, a test kit provided by ELITechGroup (Puteaux, France). A Selectra Pro XL device (Puteaux, France, 2016) at the Provincial Laboratory was used for the HbA1c measurements. All procedures were performed according to the manufacturer’s brochures and guidelines.

### Linear regression analysis

First, a multiple linear regression model was established to predict HbA1c levels, incorporating covariates such as age, gender, education, BMI, past medical history of hypertension, current smoking status, and scores from ADL and IADL assessments. The multiple linear regression model can be formulated as Eq. (1):

1 $$Y= {\beta }_{0}+{\beta }_{1}{X}_{1}+{\beta }_{2}{X}_{2}+\dots +{\beta }_{n}{X}_{n}+\varepsilon$$

in which $Y$ is the level of HbA1c, ${\beta }_{0}$ is the intercept, ${\beta }_{1}$ to ${\beta }_{n}$ are coefficients, ${X}_{1}$ to ${X}_{n}$ are the covariates, and $\varepsilon$ denotes the error term. Following the statistical elimination of non-significant covariates, individual domains of MoCA-B, ADL and IADL batteries were added to the model. After investigating interactions between domains and multicollinearity, a simplified model was derived as shown in Eq. (2):

2 $$Y= {\beta }_{0}+{\beta }_{1}X+\varepsilon$$

where $Y$ is the level of HbA1c, ${\beta }_{0}$ is the intercept, ${\beta }_{1}$ is the coefficient of the significant domain $X$.

### Intra- and inter-battery correlation heatmaps

To propose a robust multiple linear regression model with battery domains as independent variables, multicollinearity of domains was investigated using heatmaps illustrating the intra- and inter-battery correlations. Redundant covariates were subsequently removed to reach Eq. (2). Also, as it is discussed in the next sections, the intra- and inter-battery correlations may provide useful information for clinicians regarding the redundancy of the MoCA-B and IADL domains. The correlation heatmaps were produced using Pearson’s *r*, as previously described for other battery correlations [24].

### Statistical analysis

Narrative statistical analysis reports the mean ± standard deviation for normally distributed variables, and median (interquartile range) for skewed distributions. All frequencies are reported as percentages. The independency of cases and controls was investigated by statistical testing of overall demographic and clinical variables. Student’s *t*-test was performed for normally distributed variables with equal variances, while Welch’s test was preferred in the case of heteroscedasticity. To compare percentages, the chi-squared test of independence was chosen. All tests were done with a two-tailed assumption and an alpha of 0.05 with 95% confidence intervals (CI). The targeted power of tests was set to be 80%, and underpowered deviations identified through post hoc power calculations are separately reported in the respective legends. Odds ratio (OR) analyses were done in two-by-two tables with adjustments of covariates. All analyses were done in R statistical software (version 3.6.0) and RStudio (version 1.2.1335) using “EpiStats”, “dplyr”, and “plotly” packages [25, 26].

## Results

### Narrative characteristics of T2DM patients

60 T2DM patients were included based on the inclusion/exclusion criteria from whom one-half had a measurement of HbA1c more than 8%, i.e., cases, at the time of the study. The mean of HbA1c of cases was 10.12% and that of controls was 7.32%. The mean age ± SD of all 60 patients at the time of their T2DM diagnosis was 52.6 ± 8.5. Other characteristics of patients have been presented in Table 1. The demographic characteristics of the two groups were similar, except for HbA1c which was the only criterion that distinguished the cases from the controls.

**Table 1:** Demographic and general characteristics of T2DM patients

Characteristics	PCDM (n = 30)	FCDM (n = 30)	*p-*value
Age, years	60.4 ± 8.1	58.5 ± 10.2	0.43
Women, %	20 (66.7)	16 (53.4)	0.12
Weight, kilograms	76.7 ± 10.5	74.5 ± 8.2	0.47
Height, meters	1.66 ± 0.07	1.65 ± 0.08	0.72
BMI, kg/m^2^	28.35 ± 3.74	27.56 ± 3.38	0.53
Education, years	0 (0–4.8)	3.5 (0–7.5)	0.12
History of HTN, %	13 (43.4)	16 (53.4)	0.27
Duration of T2DM, years	7 (6–9)	7 (4.5–9)	0.96
Current smoking, %	8 (26.7)	10 (33.4)	0.41
MDI against SD, %	19 (63.4)	22 (73.4)	0.25
Cr, mg/dL	0.90 (0.80–1.00)	0.95 (0.90–1.10)	0.48
HbA1c, %	10.12 ± 1.14	7.32 ± 0.63	< 0.0001

Reported values are presented in three formats based on their distributions: (i) mean ± standard deviation for normally distributed variables (tested with Shapiro–Wilk normality test); (ii) median (1st quantile–3rd quantile) for non-normal distributions; or (iii) frequency (%) for variable counts. The calculated *p*-value is that of the two-tailed student’s *t*-test when the distribution meets the normality assumption; that of the Mann–Whitney Wilcoxon test when distribution violates normality assumption; and that of chi-squared for independence when frequencies are compared. *PCDM* poorly controlled type 2 diabetes mellitus patients, *FCDM* fairly controlled type 2 diabetes mellitus patients, *BMI* body mass index, *HTN* hypertension, *MDI* multiple-dose injections, *SD* single dose, *Cr* creatinine, *HbA1c* hemoglobin A1c

### No unadjusted link between MoCA-B and HbA1c

First, we investigated the difference between MoCA-B scores in cases and controls. Both groups were balanced in size (n_1_ = n_2_ = 30), heteroscedastic (Bartlett’s test, *p*-value = 0.016), and non-normally distributed (Shapiro–Wilk normality test, *p*-value < 0.001). Kruskal–Wallis rank sum test revealed a *p*-value of 0.82. Similar result was obtained from primary analysis of unadjusted OR, which revealed no significant difference (OR = 0.64; 95% CI = 0.19–2.12) in risk for poorly controlled type 2 Diabetes Mellitus (PCDM), in individuals with an exposure of cognitive decline. Table 2 demonstrates the two-by-two table for more clarification of the patient distributions in four categories.

**Table 2:** Two-by-two table to obtain unadjusted OR for MoCA core of < 27

Variables	HbA1c > 8%	HbA1c ≤ 8%	Total
MoCA < 27	18	21	39
MoCA ≥ 27	12	9	21
Total	30	30	60
Proportion exposed	0.6	0.7	0.65

The calculated unadjusted OR is 0.64 with 95% CI of 0.19–2.12, hence non-significant

### Odds ratio adjusted for age, education and IADL

Considering age as a covariate, adjusting for younger elderly patients (age > 60; n = 34) point estimate of age-adjusted OR is 0.09 (95% CI = 0–0.92; *p*-value = 0.015, see Table 3). However, in the middle-aged patients with age ≤ 60, no association was found between MoCA-B scores and HbA1c levels (*p*-value = 0.24). Surprisingly, adjusting for age (> 60), education (≤ 7 years), and IADL (> 14) suggests a protective effect of cognitive decline in PCDM (n = 22; OR = 0.00; 95% CI = 0.00–0.26; *p*-value < 0.0001, see Table 4).

**Table 3:** Two-by-two table demonstrating adjustment for the age (> 60) of the patients

	HbA1c > 8%	HbA1c ≤ 8%	Total
MoCA < 27	10	16	26
MoCA ≥ 27	7	1	8
Total	17	17	34
Proportion exposed	0.59	0.94	0.76

The aged-adjusted OR is 0.09 with 95% CI = 0–0.92; *p*-value = 0.015. The post hoc power analysis revealed an OR calculation power of 69.2% (< 80%)

**Table 4:** Two-by-two table to compute OR adjusted for age (> 60), education (≤ 7 years), and IADL (> 14)

	HbA1c > 8%	HbA1c ≤ 8%	Total
MoCA < 27	2	14	16
MoCA ≥ 27	6	0	6
Total	8	14	22
Proportion exposed	0.25	1.00	0.73

The calculated adjusted OR is 0.00 with 95% CI = 0.00–0.26, p-value < 0.0001. The post hoc power analysis was not possible as there were no nonexposed controls in the adjusted context

### Linear regression analysis of specific domains

The linear regression analysis of cognitive domains revealed a significant association between the “delayed recall” domain of MoCA-B and the HbA1c measurement (*p*-value < 0.01), represented by the regression model in Eq. 3:

3 $$HbA1c=11.26-0.65\times Delayed Recall+\varepsilon$$

in which HbA1c is in the arbitrary unit of %, 11.26 is the intercept of the linear equation and the “delayed recall” variable has a coefficient of − 0.65, and $\varepsilon$ is the error rate. The negative sign of the coefficient means that a higher score in the delayed recall domain would result in a lower expected/calculated HbA1c. Another significant association was seen in the domain of “orientation” with a negative coefficient and a *p*-value of 0.043. Nonetheless, multiple regression models were not significant involving any combination of MoCA-B, ADL, or IADL domains. Regarding IADL domains, two domains of “managing medications” and “using the phone” were revealed to be associated with the level of HbA1c with *p*-values of 0.015 and 0.023, respectively. The coefficients in both cases were negative, indicating an inverse relationship between these instrumental abilities and the HbA1c levels.

**Fig. 1:**
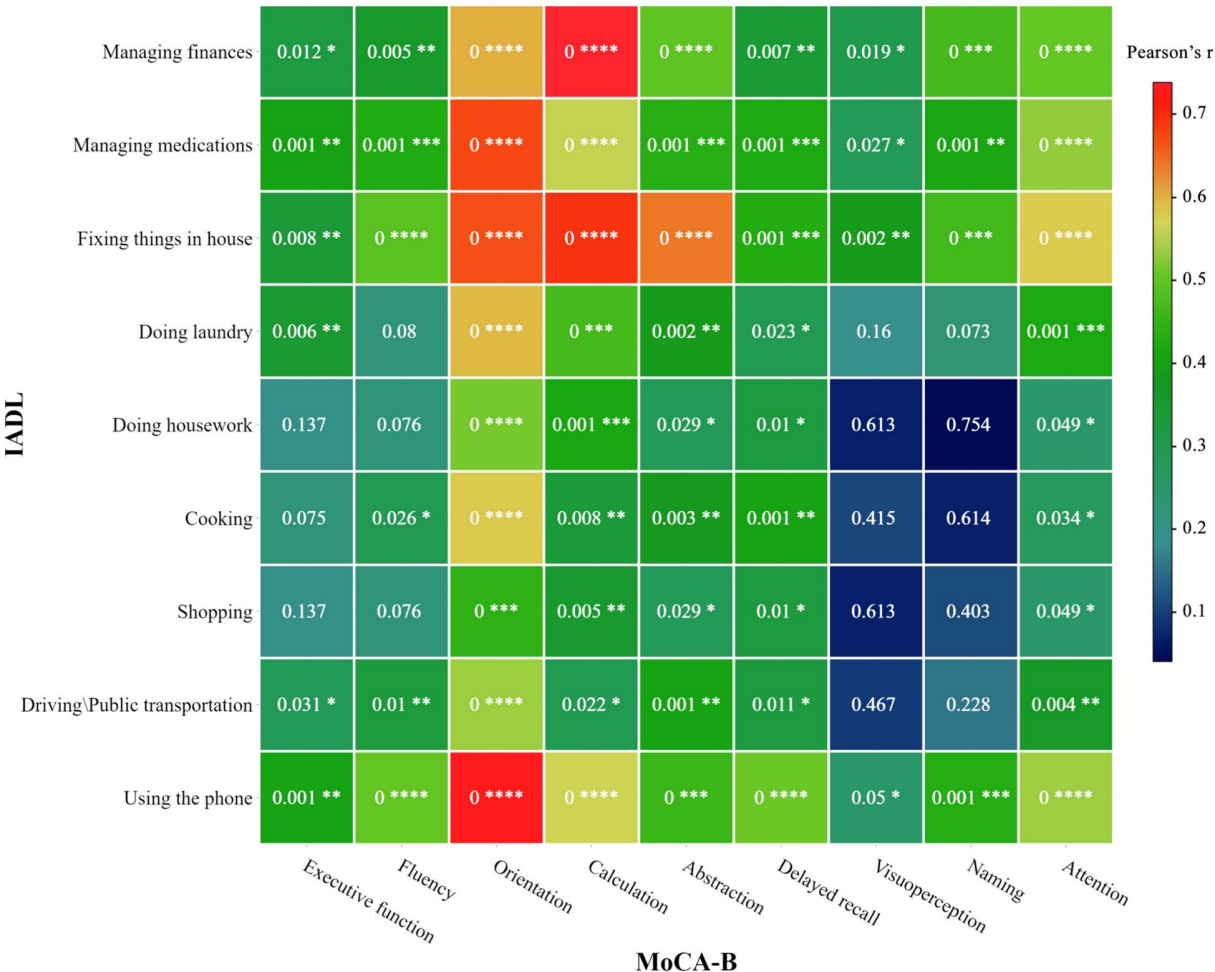
Inter-battery correlations of MoCA and IADL domains illustrated as a heatmap. Colors demonstrate the extent of correlation according to the color bar. *p*-values are written within each cell and asterisks refer to the level of significance. ****< 0.0001; ***< 0.001; **< 0.01; *< 0.05

Additionally, we investigated Pearson’s correlations in domains of MoCA-B and IADL batteries. Figure 1 demonstrates a heatmap with color-coded Pearson’s *r* and denoted respective *p*-values within each cell. As illustrated, there was a considerable correlation between the “orientation” domain of MoCA-B and the “using the phone” domain of the IADL. Interestingly, the intra-battery analysis of IADL domains suggested a strong correlation between the two domains of “managing medications” and “using the phone”. Furthermore, we found a strong correlation between the “calculation” domain of MoCA-B, and “managing finances” and “fixing things in house” domains of the IADL. The latter was also correlated with the “abstraction” domain of MoCA-B. On the other hand, “visuoperception” and “naming” domains of MoCA-B seemed to be the least correlated with IADL domains, particularly with “housework”, “shopping”, and “driving”.

**Fig. 2:**
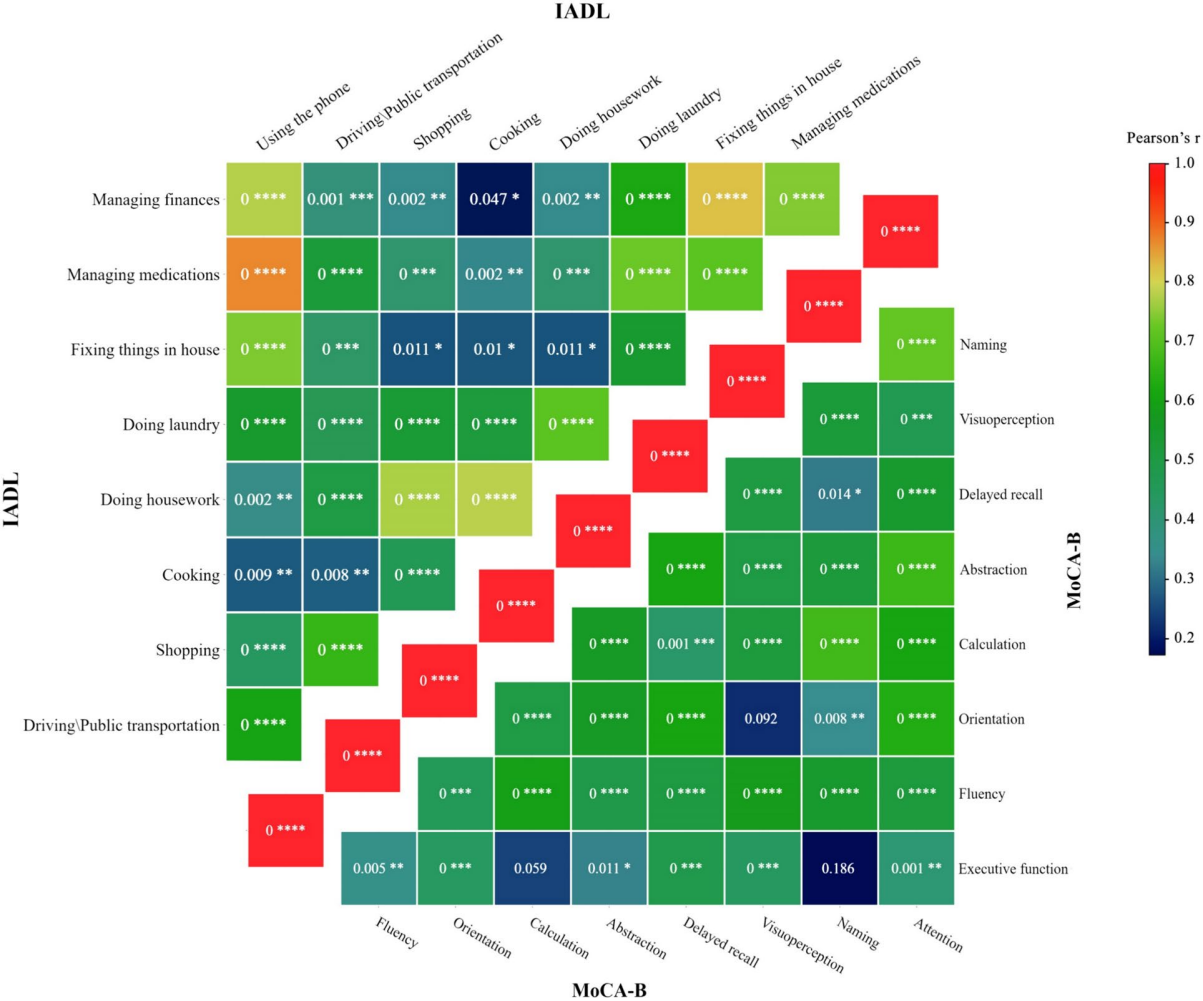
Intra-battery correlations of MoCA and IADL domains illustrated in two half-heatmaps. Colors demonstrate the extent of correlation according to the color bar. The diagonal red cells represent the correlations of identical domains. *p*-values are written within each cell and asterisks refer to the level of significance. ****< 0.0001; ***< 0.001; **< 0.01; *< 0.05

The intra-battery analysis may further help identify the (semi)-redundant domains of the batteries, considering the specific context of our study. Figure 2 demonstrates to what extent MoCA-B and IADL batteries are redundant within their domains. These correlations suggested little evidence for general redundancy between any of two domains in each battery. However, as mentioned previously, there was a noticeable correlation between the “using the phone” and “managing medications” domains of IADL. Also, “managing finances” was correlated with “fixing things in house” and “using the phone”. Similarly, “doing housework” was moderately correlated with “shopping” and “cooking” domains. Unlike MoCA-B, the IADL battery demonstrated more redundancy within its domains. Interestingly, we could not find any significant correlation between “executive function” and “naming”, nor between “orientation” and “visuoperception”, nor for “executive function” and “calculation”. The associations between domains have been further discussed in the next section.

## Discussion

Like previously reported in the literature [10, 27], we did not find any crude association between HbA1c levels and MoCA-B scores in minimally literate patients with a recent (< 10 years) diagnosis of T2DM who have also recently transitioned from oral agents to insulin injections. Evidently, this may not hold true in other populations, like elderly or patients with more than 10 years from initial diagnosis [28]. Similarly, we could not find any adjusted association between HbA1c levels and MoCA-B in middle-aged T2DM patients which is also aligned with the literature [29, 30]. Nonetheless, the OR adjusted for younger elderly, less education and high IADL suggested a surprising protective effect against PCDM. We speculate that the support of caregivers and family members in insulin administration to this patient subpopulation was an important covariate which we did not control for in this preliminary case–control study. Numerous studies have reported that managing medications by cognitively declined elderly’s family members markedly enhance adherence in chronic diseases like T2DM [31, 32]. However, other studies have reported a reversed effect of external caregivers, and not family members, on glycemic control in the elderly [33, 34].

Based on our findings, even for patients with high ADL and IADL scores, the cognitive decline measured via MoCA-B was associated with glycemic control. This finding highlights the potential importance of MoCA-B screening in T2DM compliance, compared with a screening based on ADL and IADL, especially at the time of transition to insulin injections. According to the literature, deficits in general cognition are linked to missed clinical appointments and false blood glucose readings [33, 35]. Although patients with satisfying IADL scores are well capable of administering their medications, their capability of adherence to medical visits starts to decline with cognitive deficits. Therefore, clinicians shall regardless of capability in daily activities consider cognitive function and family support in their treatment strategy. However, due to time constraints, it can be challenging for clinicians, and especially subspecialists, to screen every person with diabetes for cognitive functioning. The challenge arises when cognitive decline interferes with glycemic control, and subsequently, clinicians have to sort out the cognitive domains involved [36]. The intra-battery and inter-battery correlation analysis performed in this study helps such clinicians identify probable cognitive issues with a couple of simple questions. According to linear regression analyses, “delayed recall” and “orientation” are the most important among MoCA-B domains for good glycemic control in patients with T2DM. Among IADL domains, “managing medications” and “using the phone” are the most prominent. Based on our inter-battery analysis, “orientation” is highly correlated with “using the phone”. Also, “delayed recall” is moderately correlated with “using the phone”. Hence, it seems that a simple question of “can you work with your cellphone?” would provide the most relevant information of patient compliance in the least time. Besides, “managing medications” is highly correlated with “using the phone” according to the intra-battery Pearson’s correlation analysis of IADL. The “managing medications” domain demonstrates the second-highest relevance to MoCA-B’s “orientation” domain. This suggests that the second most crucial clinical question to assess patient compliance would be, “can you manage your medications?” While this study highlights the importance of the “delayed recall” domain in glycemic control, the “executive functioning” domain was previously demonstrated to be crucial in performing a complex insulin injection therapy [37, 38].

### Limitations

The primary limitation of the current study was the small sample size, derived from similar regional investigations [14], which led to some underpowered findings that could not be reported. The small sample size in this preliminary study underscores the need for cautious interpretation of the results and necessitates future studies to replicate our findings. Furthermore, while the investigated covariates in this study aligned with those in the literature [39, 40], the absence of other potential confounding factors might have affected the inferred association between cognitive decline and PCDM. Access to covariates like family/care giver status, social support, mood, anxiety, psychosocial functioning, intelligence quotient (IQ), hypo- or hyperglycemia history and detailed medication history could well extend the results. Also, due to financial limitations, we could not include a broad spectrum of laboratory assays. Obviously, including the measurement of the pro-inflammatory factors and lipid status could provide a deeper view on the details of the association between the glycemic control and cognitive decline. It is recommended that future studies use a larger sample size and include more covariates in their analysis.

## Conclusions

Aligned with the existing literature, this study does not find any association between MoCA-B scores and HbA1c levels in recently diagnosed middle-aged population. However, surprisingly, we found cognitive impairment results in improved glycemic control in less educated younger elderly. Limitations of the current study does not allow a concrete reasoning of this finding, leading to speculation about the potential significance of support from family members within this subpopulation. Future studies should include a larger sample size and a broad spectrum of psychosocial factors as covariates.

Moreover, within cognitive domains, we found the strongest associations between the “delayed recall” and “orientation” domains and HbA1c levels. Among IADL domains, “using the phone” and “managing medications” illustrated significant negative correlations with HbA1c levels. We further found that there is a significant correlation between MoCA-B’s “orientation” and IADL’s “using the phone” domains. Also, intra-battery analysis results revealed a strong correlation between “using the phone” and “managing medications” domains. These results suggest a potential rapid screening system in the clinics by asking about the ability of patient to use a cell phone or manage medications.

## Data Availability

The dataset generated and analyzed during the current study is available from the corresponding author on reasonable request.

## Abbreviations

ADA: American Diabetes Association
ADL: Activities of daily living
BMI: Body mass index
CBC: Complete blood count
CI: Confidence interval
DM: Diabetes mellitus
FCDM: Fairly controlled diabetes mellitus
HbA1c: Hemoglobin A1c
IADL: Instrumental activities of daily living
MDI: Multiple dose injections
MoCA-B: Montreal cognitive assessment—basic
OR: Odds ratio
PCDM: Poorly controlled diabetes mellitus
SD: Single dose
T2DM: Type 2 diabetes mellitus
